# Simulating the Skin Permeation Process of Ionizable Molecules

**DOI:** 10.1021/acs.jcim.4c00722

**Published:** 2024-06-25

**Authors:** Magnus Lundborg, Christian Wennberg, Erik Lindahl, Lars Norlén

**Affiliations:** † SciLifeLab, ERCO Pharma AB, 171 65 Solna, Sweden; ‡ Department of Applied Physics, SciLifeLab, KTH Royal Institute of Technology, 106 91 Stockholm, Sweden; § UC AB, 111 64 Stockholm, Sweden; ∥ Department of Biophysics and Biochemistry, SciLifeLab, Stockholm University, 106 91 Stockholm, Sweden; ⊥ Department of Applied Physics, Swedish e-Science Research Center, KTH Royal Institute of Technology, 106 91 Stockholm, Sweden; # Department of Cell and Molecular Biology (CMB), Karolinska Institutet, 171 77 Solna, Sweden; ∇ Dermatology Clinic, Karolinska University Hospital, 171 77 Solna, Sweden

## Abstract

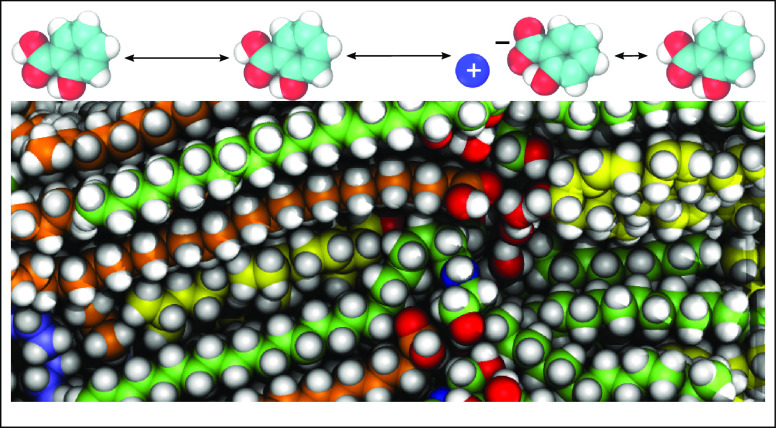

It is commonly assumed that ionizable molecules, such as drugs, permeate through the skin barrier in their neutral form. By using molecular dynamics simulations of the charged and neutral states separately, we can study the dynamic protonation behavior during the permeation process. We have studied three weak acids and three weak bases and conclude that the acids are ionized to a larger extent than the bases, when passing through the headgroup region of the lipid barrier structure, at pH values close to their p*K*_a_. It can also be observed that even if these dynamic protonation simulations are informative, in the cases studied herein they are not necessary for the calculation of permeability coefficients. It is sufficient to base the calculations only on the neutral form, as is commonly done.

## Introduction

Understanding and eventually accurately predicting the skin permeation process are important challenges when developing drugs and drug formulations for topical or transdermal drug delivery. It has been shown that, for most permeants, the extracellular lipids in the stratum corneum (SC) provide the main permeation barrier^1−4^ while polar compounds might follow complementary permeation pathways, such as through corneocytes.^5,6^ The viable epidermis may also provide a significant permeation barrier for very lipophilic molecules.^7^ Still, the extracellular lipid matrix in stratum corneum is recognized as the primary permeability barrier, and it is also a barrier that is possible to modulate using chemical permeation enhancers^5^ to allow much broader classes of drugs to be delivered with transdermal patches.

With the term ionizable compounds, we refer to weak acids and bases, i.e., molecules with pH-dependent protonation states. It is commonly assumed that only the neutral form passes through biological lipophilic membranes, which is often referred to as the pH-partition hypothesis.^8^ This approach can provide a quick estimate of the effect of the pH on the permeation of ionizable molecules. However, it is a simplification that ignores the possibility of permeants changing their ionization states during the permeation process. This is particularly likely since the environment during permeation changes several times between aqueous, zwitterionic, or charged lipid headgroups and purely hydrophobic regions. On a microscopic level, it is possible to study this type of process by using molecular dynamics (MD) simulation extended with dynamic protonation, which has been applied, e.g., to study the permeation of propranolol through a 1-palmitoyl-2-oleoyl-sn-glycero-3-phosphocholine (POPC) bilayer.^9^ By using umbrella sampling simulations combined with either a constant-pH protocol or simulations of the neutral and positively charged forms separately, good agreement was observed between permeability coefficients calculated using the lowest free energy path from the combination of the fixed-ionization-state of charged/neutral forms and those based on the constant-pH method.^9^

**Figure 1:**
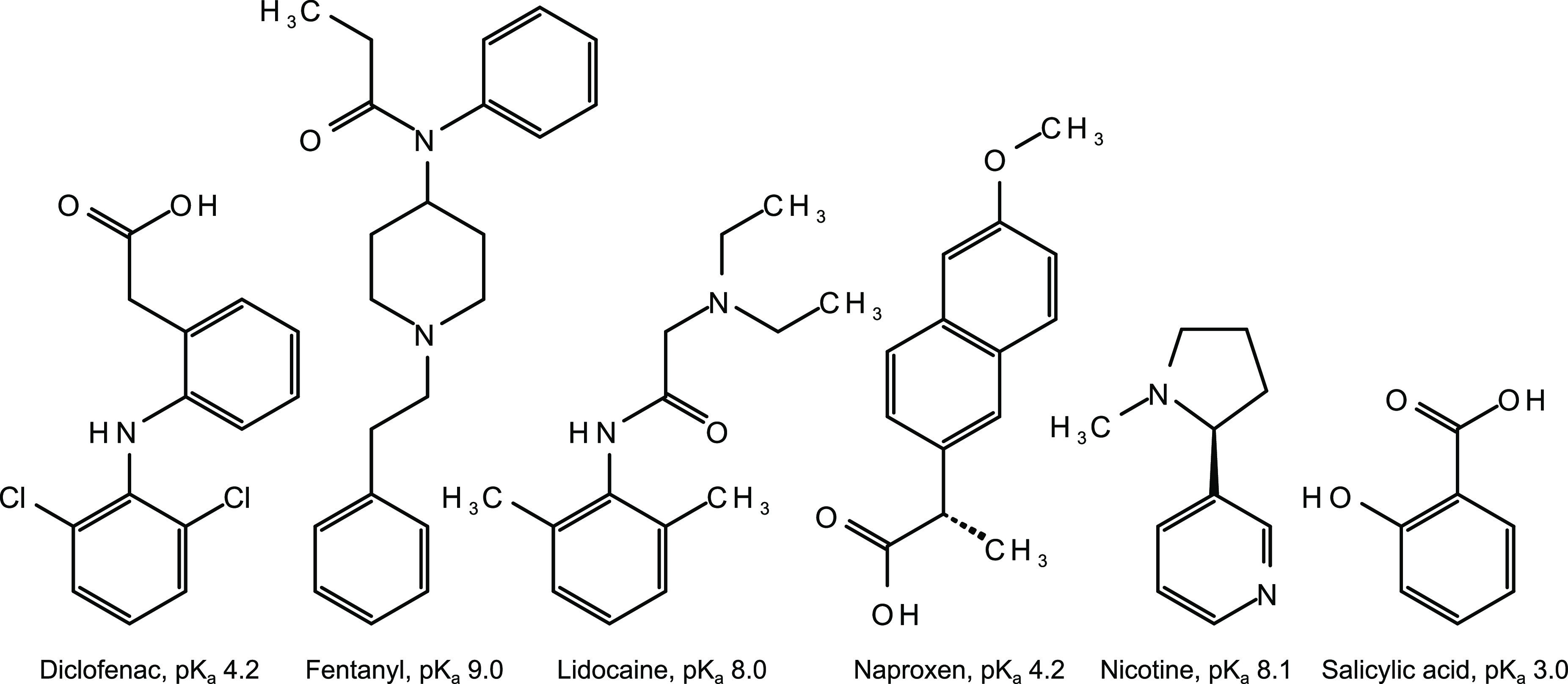
Six molecules are included in this study. The p*K*_a_ values of diclofenac, fentanyl, lidocaine, and naproxen are from Settimo et al.^17^ Values for diclofenac and naproxen were also obtained from Packer et al.^18^ The p*K*_a_ of nicotine is from Banyasz,^19^ and the salicylic acid p*K*_a_ is from Serjeant and Dempsey.^20^

We have previously developed an atomistic model of the stratum corneum lipid barrier, based on near-native cryo-electron microscopy (cryo-EM) images and MD simulations followed by cryo-EM image simulation.^10^ The lipid barrier model consists of an equimolar mixture of ceramides, cholesterol, and free fatty acids. Ceramides are of types NS, NP, and acyl ceramide EOS. The acyl chain lengths of ceramides NS and NP, as well as those of free fatty acids, range from 20 to 30 carbons with a realistic distribution.^11^ The ceramides are in a splayed configuration. Cholesterol is primarily, but not exclusively, located in the sphingoid chain region. The free fatty acids are in only the fatty acyl chain region. There is one water molecule per lipid molecule in the system, corresponding to a water concentration of approximately 0.65 M in the lipid structure, and they are located in the lipid headgroup region. We have shown that the lipid barrier model can be used for predicting the skin permeability coefficient of a wide range of permeants^12,13^ and to help understanding the function of chemical permeation enhancers in topical formulations.^14^ In this study, we have used the same atomistic model to evaluate if it can also be used for predicting the skin permeability of ionizable molecules with dynamic protonation. Six molecules have been selected (see Figure 1), three weak acids and three weak bases, each with previously published human skin permeability coefficients in a range of pH values. As mentioned above, there are two primary MD simulations methods available for studying the permeation process of ionizable molecules: The first one is truly constant-pH simulations, in which the protonation state of the molecule is allowed to change throughout the simulation depending on the nature of its surroundings. The second method is to run two separate simulations, for the uncharged and charged states, and then combine the results based on the free energy difference between the two states throughout the membrane. The first method is, in principle, elegant in that the change in protonation state can be captured from a single simulation setup—multiple replicas are still required. However, one simulation is required for each pH-value of interest, and the methodology is still quite new and can require new parametrization of molecules.^15,16^ The second method needs two simulations but is independent of pH by recalibrating the output potentials of mean force (PMFs), or free energy profiles. In this work, we have used the second method since we have examined more than two pH values for each permeant. To fully capture the chemical reactions, such as proton exchange, that occur during the permeation process, QM/MM (quantum mechanics/molecular mechanics) MD simulations can be used. However, such methods would require more computational resources.

Apart from the pH-partition hypothesis,^8^ there are other empirical methods to calculate the permeability coefficient or flux of ionizable molecules.^21−23^ These equations provide a quick estimate of the permeability of neutral, ionic, and partially ionized compounds, but they do not yield a deeper understanding of the permeation process itself.

The permeation process through the skin’s lipid barrier structure is complex compared to most other biological membranes. First, there can be other permeation pathways that are more favorable for very polar or ionizable molecules, but all molecules are expected to pass through the lipid barrier structure to some extent. Second, it is difficult to know what the actual pH is inside the lipid barrier structure given its very low water contents. The surface of the skin has been shown to be slightly acidic, with a buffer capacity and pH in a range of 4 to 6.^24,25^ There is a sigmoidal pH gradient across the stratum corneum that reaches approximately 7 in the bottom layers.^24,26,27^ This means that the pH of the donor solution might not suffice to accurately decide what pH is relevant when calculating the permeability coefficient. This becomes even more apparent when simulating the skin permeation of ionizable molecules from an aprotic solvent. Such fundamental problems need to be kept in mind, but we still believe that the methods and results presented herein can provide an important additional understanding of the skin drug permeation process.

## Methods

The results (PMFs and diffusion coefficient profiles) from simulations with molecules in their uncharged state are from ref (13). For completeness, those results are included in the data associated with this article; see the Data and Software Availability section below.

The molecular dynamics simulations, of molecules in their charged state, were run using GROMACS 2022,^28−30^ with a source code modification to enable symmetrizing the accelerated weight histogram (AWH) sampling along a spatial reaction coordinate. These changes are available from the GROMACS gitlab repository.^31^

van der Waals interactions had a cutoff of 1.2 nm with a smooth force-switch from 1.0 to 1.2 nm. Coulomb interactions were calculated using PME^32^ with a radius of 1.2 nm. Bonds to hydrogen atoms were constrained using the P-LINCS algorithm.^33,34^ TIP3P^35^ parameters were used for water molecules. For the lipid molecules, the CHARMM36 lipid force field^36,37^ was used, without dispersion corrections for energy or pressure. Ceramide parameters were modified to more accurately reproduce the ceramide NP crystal structure,^38^ as described in ref (10). In order to allow a 3 fs integration time step, hydrogen atoms were made three times heavier by repartitioning the corresponding mass from their bound heavy atoms.^39^ The temperature was set to 305.15 K by using a stochastic dynamics integrator^40^ (also referred to as a velocity Langevin dynamics integrator) with a time step of 3 fs and with a time constant τ of 2 ps (corresponding to a friction constant of 0.5 ps^–1^). The pressure was set to 1 atm and controlled using a stochastic cell rescaling barostat^41^ with a time constant of 1.0 ps and a compressibility of 4.5 × 10^–5^ bar^–1^. Semi-isotropic pressure coupling was applied to allow the barrier system to expand/contract laterally, while the spacing in the normal (*Z*) dimension was kept constant by setting the compressibility to zero.

Alchemical reaction coordinates were sampled using AWH for (de)coupling the solute in water or in the lipid barrier system.^42−44^ The interactions were decoupled using 21 equidistantly distributed λ states, decoupling van der Waals and Coulomb interactions simultaneously. Soft-core transformations^45^ with α = 0.5 and σ = 0.3 nm were applied to both the van der Waals and Coulomb interactions of the solute.

Topologies, i.e., inter- and intramolecular interaction parameters, for all permeants and formulation components except water, were generated using STaGE,^46^ which in turn uses Open Babel^47^ and MATCH^48^ to generate GROMACS topologies compatible with the CGenFF^49^ CHARMM force field.

When performing simulations with charged molecules, either in solvent or in the skin’s barrier structure, counterions were (de)coupled together with the molecule in order to keep the system net neutral. Sodium was used as a counterion together with the weak acids (diclofenac, naproxen, and salicylic acid) in their charged state, whereas chloride was used with the weak bases (fentanyl, lidocaine, and nicotine). A flat-bottomed distance restraint potential was used to keep the ion from drifting too far beyond the electrostatic cutoff distance of the charged group. The force was set to scale from 0 to 1000 kJ mol^–1^ nm^–2^ over a distance of 1.3 to 2.5 nm. The use of a counterion, especially with a distance restraint, is admittedly artificial. In solution, the counterion would be solvated and further away from the permeant than in the skin’s barrier structure, in which there is very little water available, especially in the lipid tail regions. In order to study the effect of different counterion treatments, we also tested simulating charged diclofenac using an inverse distance restraint potential to its counterion with a force scaling from 1000 kJ mol^–1^ nm^–2^ to 0 over a distance from 0 to 1.8 nm, as well as spreading the counter charge over 50 random water molecules in the system and also excluding the counter charge completely, with possible artifacts from a net charged system. The results are presented in Figure S1 in the Supporting Information.

After having performed the simulations, we noticed that the AWH reaction coordinate dimension, which samples states along the spatial position of the permeant across the barrier structure, acted on the center of mass of the permeant molecule together with its counterion instead of only on the permeant itself. This only affected the results of the charged molecules and is expected to have resulted in a slightly smoother PMF, especially lowering the highest peaks due to the higher inherent flexibility in the center of mass of the molecule together with its counterion. With the very high free energy barriers, we do not expect this to have affected the final permeability coefficients in any significant way.

### Hydration Free Energy Calculations

The hydration free energy of each molecule was calculated by inserting it into a waterbox at a random position with its interactions with the surroundings turned off, as if in a vacuum. An input AWH diffusion constant of 1 × 10^–3^ ps was used for the hydration free energy calculations, which means that it is estimated to take approximately 1 ns to cross the alchemical dimension for one AWH walker. The AWH initial error was set to 10 kJ mol^–1^. Sixteen communicating AWH walkers were run in parallel, with the requirement that only simulations that covered the whole alchemical reaction coordinate counted toward the covering check in the initial AWH stage. The simulations were 60 ns long per walker for a total simulation time of 960 ns per solute. For a more detailed description of alchemical hydration free energy calculations using AWH see refs (13) and (44).

### Skin Barrier System Permeability Calculations

The permeability coefficient through 30 ± 6^13^ layers of the lipid barrier, *K*_P_, and the permeation resistance, *R*, were calculated as follows:^50^
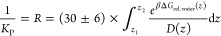
1where Δ*G*_rel.water_ is the free energy relative to the hydration free energy. When calculating the permeability coefficients, an additive constant of each PMF was chosen so that the PMF was never below 0.^13,51,52^

The local diffusion coefficient *D*(*z*) across the barrier is estimated from the friction metric *g*(*z*) calculated in the AWH simulation,^53^ via an Einstein relation *D*(*z*) = *g*^–1^(*z*). More details are available in ref (13).

**Figure 2:**
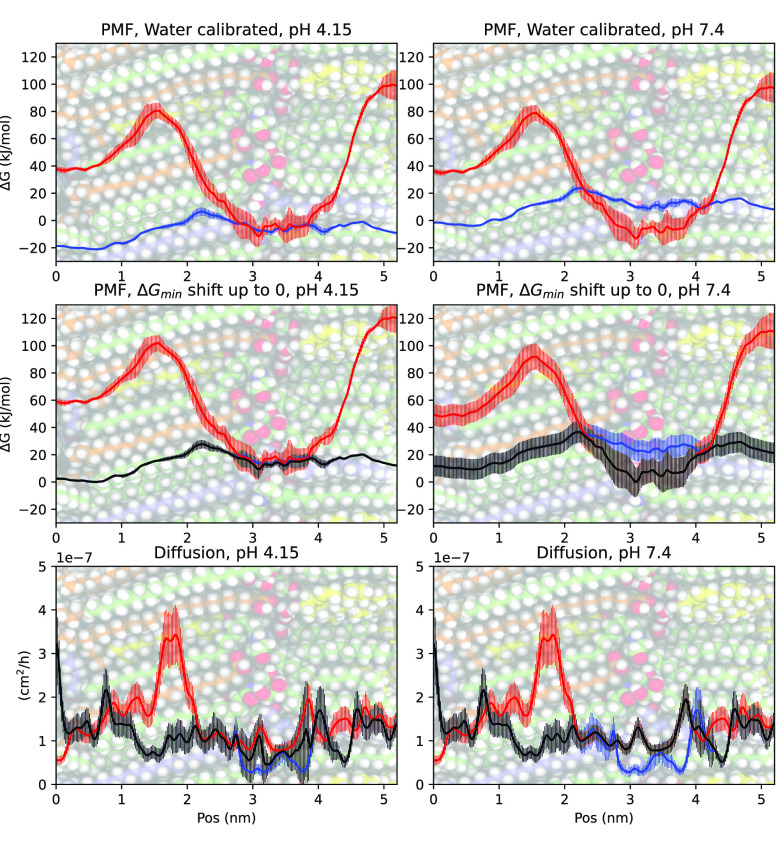
Calculation of dynamic protonation PMFs and diffusion coefficients from the charged and uncharged states. In this example, data from diclofenac is shown. In the top row, the PMFs of the neutral (blue) and charged (red) states are presented. The PMFs are calibrated relative to the hydration free energy of the two states, adjusted according to the pH – p*K*_a_ difference, at pH = p*K*_a_ (left column) and pH = 7.4 (right column). In the middle row the PMFs have been shifted upward so that the absolute minimum is ≥0, keeping the same relative free energy difference between the charged and neutral states. In the middle row the black profile shows the combined free energy profile, corresponding to the probability of being in either of the two states. In the bottom row the corresponding diffusion coefficients are shown, based on the relative distribution of charged and neutral states. The presented error bars correspond to 1 SEM (standard error of the mean).

The permeability coefficients were obtained by numerical integration across a whole bilayer with a point spacing of approximately 0.01 nm. The PMFs were symmetric across the system, and in Figures 2 and 4 only one-half of the PMFs across the lipid bilayer is shown. This means that *z*_1_ ≃ −5.2 nm and *z*_2_ ≃ 5.2 nm.

To reduce the noise of the local diffusion coefficient curves, a 0.2 nm wide rolling median filter was applied. When symmetrizing the sampling, along the spatial dimension, by using the absolute coordinate values and accounting for the AWH bias across the sampling boundaries, there are usually artificial spikes at the edges of the PMFs. These were removed by setting the two lowest (0.005 and 0.015 nm) and highest (5.200 and 5.210 nm) points in the PMF to the value of their neighbors (0.025 and 5.19 nm, respectively). These minor adjustments had no effect on the calculated permeability coefficients.

At the start of each AWH walker simulation, the permeating molecule was inserted in the lipid barrier structure at a random position with all interactions with its environment turned off. The free energy profile through the skin’s barrier structure was calculated using a two-dimensional AWH setup, using a harmonic potential to steer the permeant across the system, also referred to as the *Z* dimension, and an alchemical free energy reaction coordinate.^44^ This allows sampling the free energy along the permeation direction and also the relative insertion free energy of the permeant from the vacuum. In turn, this enables a direct calibration to the hydration free energy since the vacuum state is the same in both cases. After calibration, each point in the PMF corresponds to the free energy of transfer from the water vehicle to that point of the lipid barrier structure. Like when calculating the hydration free energy, the estimated AWH initial error was set to 10 kJ mol^–1^. The AWH input diffusion constant was set to 3 × 10^–5^ nm^2^ ps^–1^ for the spatial pulling dimension and 5 × 10^–5^ ps^–1^ along the alchemical free energy dimension. The input diffusion constant affects only the AWH histogram size; it does not affect the computed diffusion coefficient, which is obtained from the AWH friction metric during data analysis. The AWH force constant along the spatial *Z* dimension (normal to the lamellar stack), steering the permeant relative to the ceramide fatty acid chains using a harmonic pull potential, was set to 25 000 kJ mol nm^–1^. The force constant also determines the resolution along the reaction coordinate dimension. For each permeant, five sets of simulations were run with heavy hydrogen atoms (see above) and a 3 fs integration time step. These were run using 24 communicating walkers, each running for 450 ns. The covering check in the initial AWH stage took into account simulations that covered only the whole alchemical dimension and at least a diameter of 0.8 nm along the spatial dimension. From these simulations, a combined diffusion coefficient was calculated using the AWH friction metric from all contributing walkers. The combined PMF was derived from the average of the independent PMFs from the five sets of simulations.

Along the alchemical free energy dimension, it is the end states, i.e., the fully interacting and fully decoupled states, that are of highest interest, as the difference in free energy between them corresponds to the probability of transferring the permeant from vacuum into the skin’s barrier structure. Therefore, the target distribution used in these simulations put more weight on the end states, especially the state with interactions fully turned on.^13^ Along the spatial pulling dimension, the target distribution was uniform.

The large free energy differences along the alchemical reaction coordinates of charged permeant molecules mean that long simulation times are required to obtain a sufficient AWH bias potential to allow efficient sampling of the free energy landscape. To mitigate that problem, we first performed a quick screening of the free energy landscape to use as an input along the alchemical reaction coordinate. Those screening simulations were only 15 ns long but with the AWH initial error and diffusion constant along the spatial dimension set twice as high as in the production simulations, efficiently increasing the initial update size by a factor of 8. The rough approximation of the free energy difference, along the alchemical reaction coordinate, in the headgroup region was used as an input PMF to the AWH production simulations.

### Permeability Calculations with Dynamic Protonation

After having calculated the PMFs and diffusion coefficients of the permeant molecule in its charged and neutral states, a combined, dynamic protonation PMF can be constructed. This can be done using the following procedure, (see also Figure 2):

1. Shift the two PMFs, based on the pH and p*K*_a_, according to the Henderson–Hasselbalch equation (blue and red curves in Figure 2, top row):
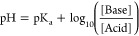
2
2. Shift both PMFs by the same amount, so that Δ*G*_min_ ≥ 0 (blue and red curves in Figure 2, middle row). This does not affect the relative difference between the PMFs but is necessary when calculating the permeability using eq 1.
3. Make a combined PMF (Δ*G*(*z*)), based on the probability sum of being in either the charged or neutral state (black curve in Figure 2, middle row). This is the same as the probability weighted average of the two PMFs.
4. Make a combined diffusion coefficient profile (*D*(*z*)), based on the probability weighted average of the diffusion coefficient profiles (black curve in Figure 2, bottom row), with the probability based on the corresponding PMFs.
5. Calculate the permeability coefficients using eq 1 and the combined Δ*G*(*z*) and *D*(*z*) profiles.

## Results and Discussion

**Figure 3:**
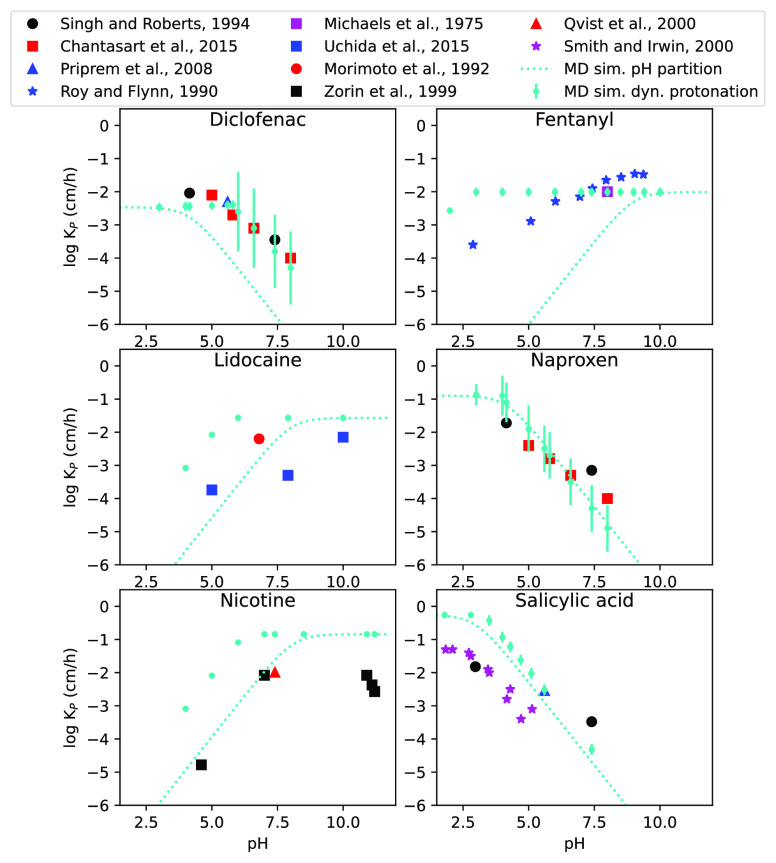
Correlations between the calculated permeability coefficients and experimental data. The cyan markers show the dynamic protonation results from MD simulations of the neutral and charged states, with error bars representing the standard error. The cyan dotted lines show permeability coefficients according to the pH-partition hypothesis^8^ based on the calculated permeability coefficient of the neutral state. The experimental values are from Singh and Roberts,^54^ Chantasart et al.,^55^ Priprem et al.,^56^ Roy and Flynn,^57^ Michaels et al.,^58^ Uchida et al.,^59^ Morimoto et al.,^60^ Zorin et al.,^61^ Qvist et al.,^62^ and Smith and Irwin.^63^

Figure 3 shows the comparisons between the calculated and experimental permeability coefficients at different pH values. It can be observed that the general trends of the experimental data are captured by the results from the simulations of the neutral and ionized states of the molecules.

From Figure 3 it is apparent that the calculated diclofenac and naproxen permeability coefficients agree best with experimental observations. Salicylic acid seems to perform fairly well, but it overestimates the permeability, at least at low pH. Comparing the calculated permeability coefficients from the charged and uncharged states as well as the results using the pH-partition hypothesis^8^ to the experimental values it is apparent that the second alternative is at least as good; i.e., the cyan dotted lines fit the experimental data at least as well as the cyan markers in Figure 3. It is only diclofenac that seemingly gains from using the dynamic protonation protocol. Fentanyl also seems to be slightly improved, but the calculated values and experimental data still do not agree very well.

Some of the large deviations between simulation data and *in vitro*/*ex vivo* data may be attributed to the classical biophysical force field used herein. It is possible that it cannot fully represent all permeants and lipid barrier molecules, accurately enough to model the permeation process in full detail. We have seen before that similar MD simulation permeability calculation methods, using just the pH-partition hypothesis instead of taking protonation states into account, give good results, but also that there are outliers.^13^ The effect of polarization during the permeation process is not taken into account by using this force field. Perhaps the simulations could be even more accurate if a polarizable force field, such as one using a Drude oscillator model,^64^ was employed.

In the Supporting Information, the PMFs and diffusion coefficients of the uncharged and charged states are shown in Figures S2 and S3. As expected, those PMFs show that the charged states of the permeant molecules have a much higher probability of visiting the polar headgroup region than the lipid side chains, especially in the region where they are tightly packed. The neutral states of the molecules are significantly less hydrophilic and have a relatively higher preference to stay in the lipid chain region.

## Conclusions

We have shown that combining MD simulations of charged and uncharged states can give a better understanding of how ionizable compounds permeate the skin barrier, including resolving in what regions the titration state is likely to change and how it influences local interactions and diffusion. The calculated permeability coefficients agree, in general, with published experimental results, indicating that the method itself is working. However, we emphasize that the pH-partition hypothesis, assuming that only the neutral state passes through the lipid barrier, performs at least as well for most of the cases studied here. Calculating the permeability coefficients according to that hypothesis requires only simulations of the uncharged state for each permeant and then multiplying the calculated permeability coefficients by the relative concentration of the uncharged state. Thus, for now we do not propose dynamic protonation to be the primary method when studying the skin permeability of ionizable compounds, but if a deeper understanding of the permeation process at different pH values is warranted, it is a relatively simple method to use.

Studying the skin permeability of ionizable compounds is further complicated by the fact that the actual pH in the skin barrier structure is not exactly known but is presumably close to the surface pH of 4 to 6.^24,25^ To accurately model the pH influence, it would be necessary to determine the balance between the buffer capacities of the donor solution/formulation and the skin itself or, more specifically, the skin’s barrier structure in stratum corneum. It might also be necessary to account for the polarization effects when charged compounds interact with the lipids.

Unfortunately, available experimental permeability coefficient data are insufficient to draw any clear conclusions about the calculation predictions. It would be valuable to have consistent measurements, replicated in different laboratories, in even larger ranges of pH. Fentanyl and nicotine have the largest pH spans from single experimental setups.^57,61^ If all molecules had such pH series or even more extensive, it would help significantly.

**Figure 4:**
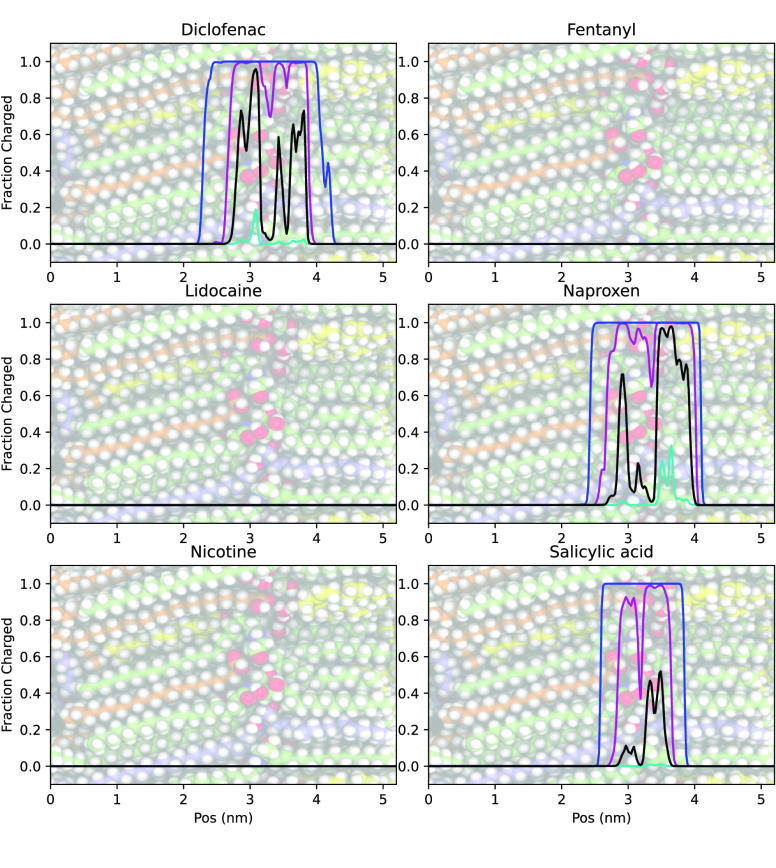
Relative amounts of the charged (ionized) state of the six studied molecules. The colors represent different pH values: black is pH = p*K*_a_, cyan is pH = p*K*_a_ – 1, magenta is pH = p*K*_a_ + 1, and blue is pH = 7.4. For Fentanyl, Lidocaine, and Nicotine all curves overlap. In the background of all plots, a representation of the lipid barrier system is shown to clarify where the headgroup region is located (2.9 to 3.3 nm).

The primary observation from the MD simulations combined into a dynamic protonation setup is that all these molecules permeate mainly, but often not exclusively, in their uncharged state (Figure 4). This is in agreement with the simulations of propranolol permeability through a POPC lipid bilayer.^9^ The charged state is predicted to affect the permeability only if its PMF minimum is below 0 and also lower than the PMF minimum of the uncharged state after both PMFs have been shifted according to the pH and the p*K*_a_ of the molecule. For example, in the presented example of diclofenac, at pH 7.4 the charged state is predicted to affect the permeability, but not at pH 4.2, according to the middle row in Figure 2.

It can also be seen that the permeability coefficients of the acidic compounds, diclofenac, naproxen, and salicylic acid, are predicted to be more sensitive to the pH of the donor solution/formulation. Under *in vitro* conditions, this would correspond to an observation of a relatively higher permeability of the ionized species of the basic compounds, since they are in fact mostly permeating in their neutral state, even if the ionized state is prevalent in solution.

## Data Availability

The input and output of the MD simulations, as well as scripts to run the analyses, are available for download from 10.5281/zenodo.11071056

## Supporting Information Available

A figure showing the effect of different ways to treat the change in net charge in the system when transforming the permeant molecules (Figure S1). Two figures showing the PMFs and diffusion coefficients of the permeants in their neutral and charged states (Figures S2 and S3) (PDF)
