# Supplementary material for: Simulating the Skin Permeation Process of Ionizable Molecules
Source: J Chem Inf Model. 2024 Jun 25;64(13):5295–302. doi: 10.1021/acs.jcim.4c00722 (PMC11234375; doi:10.1021/acs.jcim.4c00722)
Supplement: Supplementary file 1 — ci4c00722_si_001.pdf [file ci4c00722_si_001.pdf]

Supporting Information: Simulating the Skin Permeation Process of Ionizable Molecules

Magnus Lundborg,[*,†,‡] Christian Wennberg,[†,¶] Erik Lindahl,[§,‖] and Lars Norlén[*,⊥,#]

†*ERCO Pharma AB, SciLifeLab, 171 65, Solna, Sweden*

‡*Current affiliation: Department of Applied Physics, SciLifeLab, KTH Royal Institute of Technology, 106 91, Stockholm, Sweden*

¶*Current affiliation: UC AB, 111 64, Stockholm, Sweden*

§*Department of Biophysics and Biochemistry, SciLifeLab, Stockholm University, 106 91, Stockholm, Sweden*

‖*Department of Applied Physics, Swedish e-Science Research Center, KTH Royal Institute of Technology, 106 91, Stockholm, Sweden*

⊥*Department of Cell and Molecular Biology (CMB), Karolinska Institutet, 171 77, Solna, Sweden*

#*Dermatology Clinic. Karolinska University Hospital, 171 77, Solna, Sweden*

E-mail: *magnus.lundborg@scilifelab.se; *lars.norlen@ki.se

Results

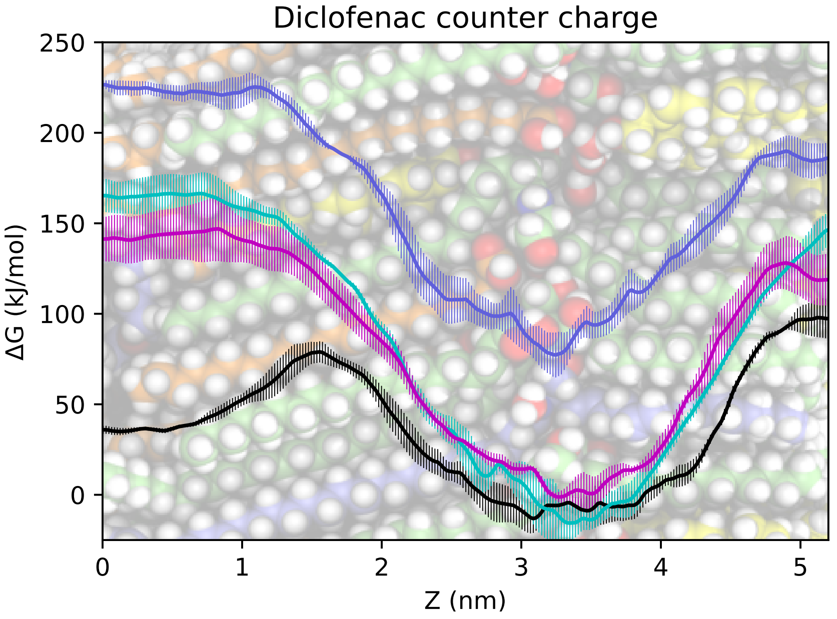

Figure S1: Effect of counter charge methods. Diclofenac PMFs using a sodium ion at short range (increasing force in range 1.3 nm to 2.5 nm) shown in black, using a sodium ion at long range (increasing force in range 1.8 nm to 0 nm) shown in cyan, spreading the counter charge over 50 water molecules shown in magenta and without any counter ion shown in light blue.

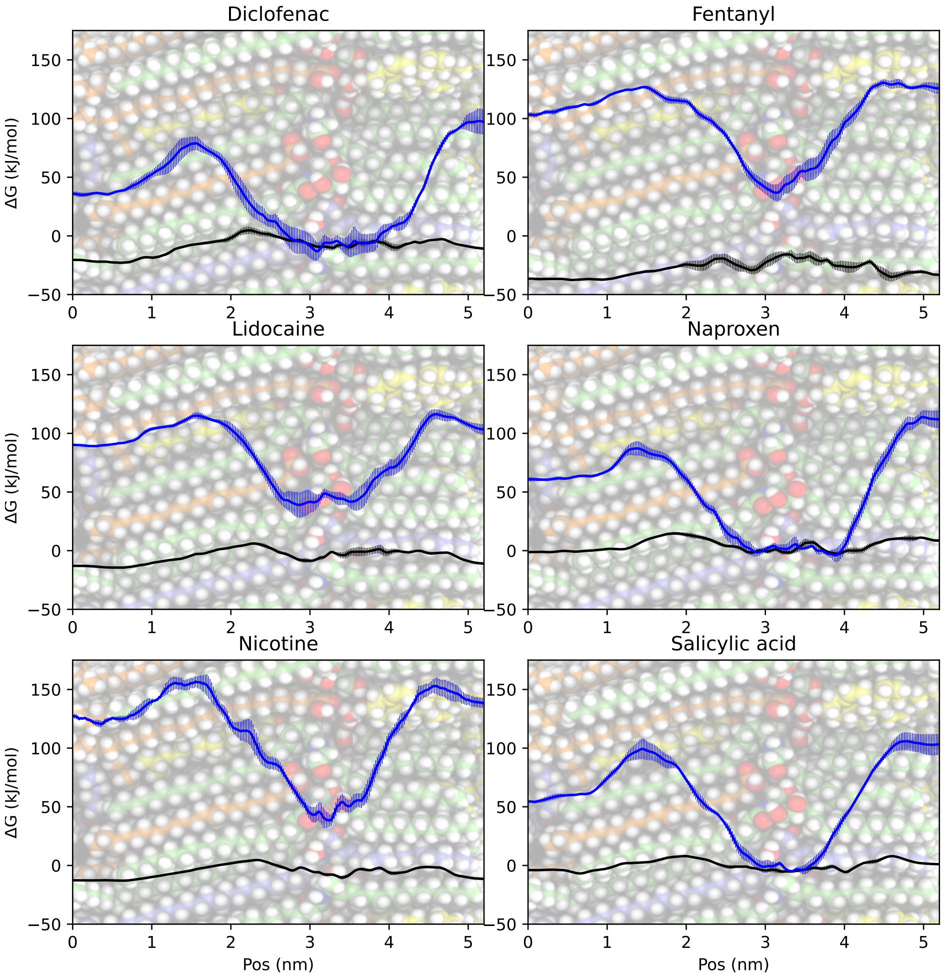

Figure S2: PMFs of uncharged and charged states of permeants. The black PMFs represent the uncharged state, whereas the blue curves show the PMFs of the charged state. The solvation free energy in water was used as reference ($\Delta G = 0$).

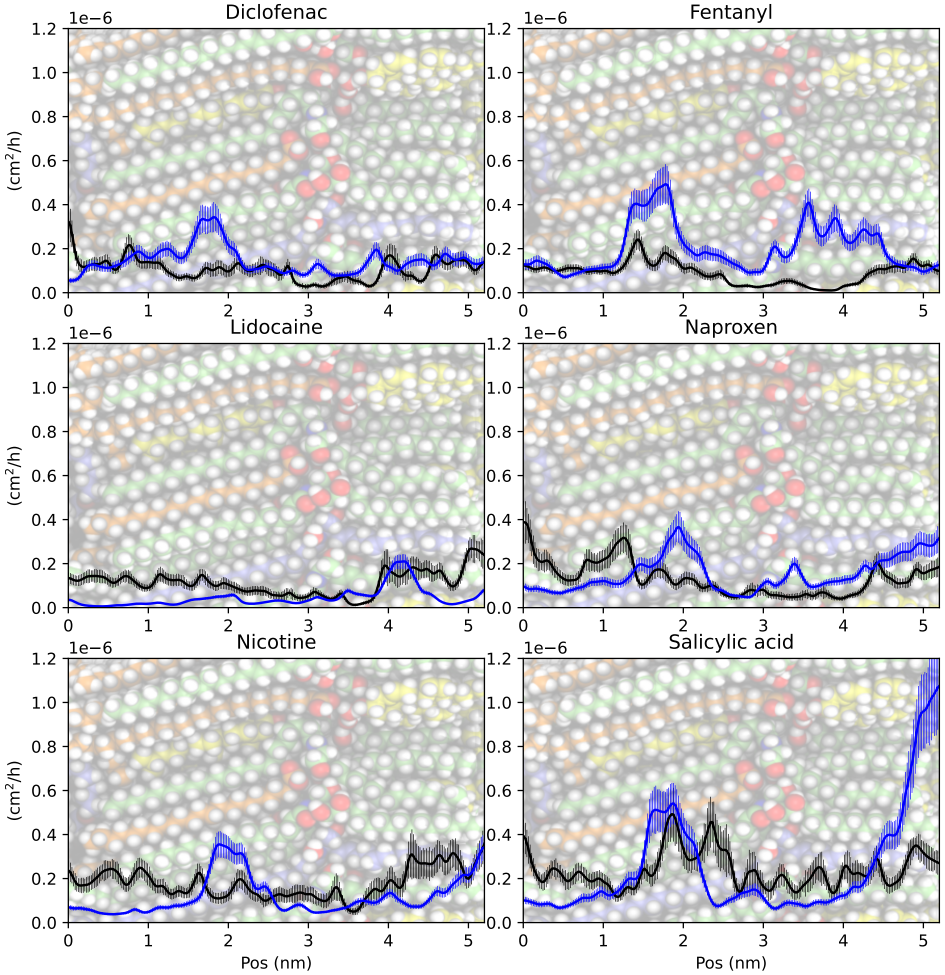

Figure S3: Diffusion coefficients of uncharged and charged states of permeants. The black curves represent the uncharged state, whereas the blue curves show the diffusion coefficients of the charged state.
